# In silico structure-based design of peptides or proteins as therapeutic tools for obesity or diabetes mellitus: A protocol for systematic review and meta analysis

**DOI:** 10.1097/MD.0000000000033514

**Published:** 2023-04-14

**Authors:** Isaiane Medeiros, Ana Júlia Felipe Camelo Aguiar, Wendjilla Medeiros Fortunato, Ana Francisca Gomes Teixeira, Emilly Guedes Oliveira e Silva, Ingrid Wilza Leal Bezerra, Juliana Kelly da Silva Maia, Grasiela Piuvezam, Ana Heloneida de Araújo Morais

**Affiliations:** a Biochemistry and Molecular Biology Postgraduate Program, Biosciences Center, Federal University of Rio Grande do Norte, Natal, RN, Brazil; b Nutrition Postgraduate Program, Center for Health Sciences, Federal University of Rio Grande do Norte, Natal, RN, Brazil; c Nutrition Course, Center for Health Sciences, Federal University of Rio Grande do Norte, Natal, RN, Brazil; d Nutrition Department, Center for Health Sciences, Federal University of Rio Grande do Norte, Natal, RN, Brazil; e Public Health Postgraduate Program, Center for Health Sciences, Federal University of Rio Grande do Norte, Natal, RN, Brazil; f Public Health Department, Federal University of Rio Grande do Norte, Natal, RN Brazil.

**Keywords:** diabetes, molecular docking simulation, overweight, peptides, protein, systematic review

## Abstract

In silico studies using dynamic simulation or molecular docking have boosted the screening and identification of molecules and/or targets in studies aimed at treating diseases such as obesity and diabetes mellitus, optimizing the development of new drugs. This study aims to describe a systematic review protocol on peptides and proteins evaluated in silico as potential therapeutic agents for obesity or diabetes mellitus.

**Methods:**

This protocol followed the Preferred Reporting Items for Systematic Reviews and Meta-analyses Protocols and was registered in the International Prospective Register of Systematic Reviews database (number: CRD42022355540). The databases to be searched will be PubMed, ScienceDirect, Scopus, Web of Science, virtual health library, and EMBASE. It will be included in silico studies that evaluate the simulation by dynamics or molecular docking of proteins or peptides involved in treating obesity or diabetes mellitus. Two independent reviewers will select studies, extract data, and assess methodological quality using the adapted Strengthening the reporting of empirical simulation studies. A narrative synthesis of the included studies will be performed for the systematic reviews.

**Results:**

This protocol contemplates the production of 2 systematic reviews to be developed focusing on obesity or diabetes mellitus.

**Conclusion:**

The reviews will enable knowledge of peptides and proteins involved in research treating these diseases and will emphasize the importance of in silico studies in this context and for the development of future studies.

## 1. Introduction

Obesity is a worldwide public health problem defined as a prevalent chronic disease associated with nutritional and metabolic disorders of multifactorial origin. This disease is characterized by a high percentage of body fat resulting from several factors related to its genesis or maintenance, such as the imbalance between caloric intake and expenditure, as well as genetic, emotional, and lifestyle factors.^[1,2]^

Data from a recent statement by the World Health Organization (WHO)^[3]^ estimate that there are more than 1 billion people with obesity worldwide, including 650 million adults, 340 million adolescents, and 39 million children, and that approximately 167 million people will become overweight or obese by 2025. These individuals are classified as overweight or obese, according to the WHO,^[4]^ when the body mass index is ≥ 25 kg/m^2^ and ≥ 30 kg/m^2^, respectively.

Obesity is associated with the risk of arterial hypertension, cardiovascular diseases, cancer, and dyslipidemia. In addition to being a relevant risk factor for diabetes mellitus (DM),^[5]^ and therefore, this association is currently recognized as diabesity.^[6]^

DM is a metabolic disorder characterized by hyperglycemia and/or insulin resistance.^[7]^ Among the types of DM, the most prevalent is type 2, occurring in about 95% of cases worldwide. Additionally, the excess adipose tissue, common in obesity, is the main factor responsible for triggering the process of insulin resistance.^[7]^

According to the international diabetes federation,^[8]^ about 642 million people will be diagnosed with diabetes in 2040. Both obesity and DM affect the health systems and the global economy through increased consumption of medications, outpatient procedures, hospitalizations, and early retirements, among other consequences.^[9]^

New technologies development and search for treatments to combat and control obesity, DM, and their complications have been encouraged by the WHO to minimize economic and health damage.^[10]^ Treating these diseases involves lifestyle changes, such as adopting healthy eating habits, physical activity, and, in some cases, the use of medications and even the indication of bariatric surgery.^[11]^ In this regard, there is a continuous search for treatments, including those based on products from natural sources, such as proteins and/or peptides aimed at replacing or complementing synthetic drugs, which have many side effects.

In this sense, in silico studies have also emerged as a promising alternative in the search and optimization of new drugs, and from the perspective of ensuring safer off-label use, allowing preliminary tests by computer simulation, in addition to promoting compliance with the reduction, replacement, and refinement, guideline (reduction, replacement, and refinement) in scientific research.^[12]^

Among the proteins and peptides involved in DM-related in silico studies, there are proteins present in cow’s milk (*β*-casein) and whey (peptides analogous to Ile-Pro-Ile–IPI),^[13,14]^ soy protein (soy glycinin),^[15]^ salmon gelatin protein and peptides,^[16]^ among others.

These proteins or peptides have shown action on the inhibition of enzymes, such as dipeptidyl peptidase IV, which is expressed on the surface of most types of cells that can potentially affect glucose regulation. Besides, they can act on the most diverse proteins, such as tyrosine phosphatase 1B,^[17]^ AMP-activated protein kinase,^[18]^ interleukin 6 and chemokine ligand 2 ^[19]^ and pancreatic lipase,^[20]^ that are proteins involved in the metabolism of both diseases, obesity, and DM.

In this context, in silico studies have provided fundamental guidelines to explain the possible mechanisms of action of protein or peptide molecules with potential application in treating obesity and/or DM.^[21]^ Computer-aided tools used in in silico studies, more precisely in drug discovery, can identify test molecules, predict efficacy, and even possible side effects, boosting the bioavailability of possible drugs.^[22]^

Furthermore, these new drugs can be identified based on target structure knowledge structure-based drug design, using docking and/or molecular dynamics or ligand-based drug design. Regarding the latter, the structure of the target is unknown, and the methods used are pharmacophore modeling, molecular similarity approaches, and quantitative structure-activity relationship.^[23,24]^ These in silico strategies boost and optimize the testing of new molecules, as they identify critical interactions with therapeutic targets that, perhaps, will be efficient in the treatment of diseases, as well as obesity and DM.

Therefore, this protocol aims to conduct the development of 2 systematic reviews addressing the use of peptides and proteins assessed in in silico studies as potential therapeutic agents for 2 diseases, namely, obesity and DM. And, although these diseases have different mechanisms and targets, the methodology used in studies with bioinformatics is common to both.

## 2. Methods

### 2.1. Protocol and registration

The protocol of this systematic review was prepared following the guidelines described in preferred reporting items for systematic reviews and meta-analyses protocols,^[25]^ available in online supplemental appendix 1, and registered with the International Prospective Register of Systematic Reviews, on September 03, 2022 (CRD42022355540), and available at: https://www.crd.york.ac.uk/prospero/display_record.php?ID=CRD42022355540.

### 2.2. Patient and public involvement

None patient and public involvement.

### 2.3. Eligibility criteria

Peer-reviewed journal articles that meet the population, exposure, and population, exposure and context (Table 1), will be included in the review. The review question is: Which peptides or proteins have been used to treat obesity or DM in the in silico studies?

**Table 1:** Elements of the research question according to the PECos strategy for the 2 systematic reviews.

Description	Abbreviation	Elements of the question
Population	P	Proteins or peptides
Exposure 1*	E1	Obesity
Exposure 2†	E2	*Diabetes mellitus*
Context	Cos	In silico studies with either molecular dynamics or molecular docking

PECos = population, exposure and context.

* Exposure 1 – It is the exposure development for Systematic Review 1, which will approach the Obesity.

† Exposure 2 – It is the exposure development for Systematic Review 2, which will approach the *Diabetes mellitus.*

#### 2.3.1. Inclusion criteria.

This review will include original articles from in silico studies that assessed either molecular dynamics or molecular docking of proteins/peptides for treating obesity or DM.

#### 2.3.2. Exclusion criteria.

It will be excluded studies exclusively in vivo or in vitro, as well as preprint, review articles, theses, dissertations, letters, conference abstracts, gray literature, and molecular dynamics studies focusing on other comorbidities.

### 2.4. Information sources and literature search

A systematic literature search will be carried out to identify peptides/proteins with potential applications in treating obesity or diabetes. A comprehensive search will be elaborated without time or language restrictions, using combinations of medical subject headings terms and EMTREE terms in the following electronic bibliographic databases: PubMed; ScienceDirect; Scopus; Web of Science; virtual health library; EMBASE. Additionally, a manual search will be carried out to insert articles that may not have been found in the above databases (Table 2).

**Table 2:** Search strategies for each database to recover to answer the systematic review question: Which peptides or proteins have been used to treat obesity or diabetes mellitus in the in silico studies?.

Database	Search strategies
PubMed, scopus, science direct, and BVS	Obesity: (Protein OR peptide) AND (“in silico” OR “computer simulation”) AND (“molecular dynamics simulation” OR “molecular dynamics” OR “molecular docking simulation”) AND (obesity) Diabetes *Mellitus*: (Protein OR peptide) AND (“in silico” OR “computer simulation”) AND (diabetes)
WOF and EMBASE	Obesity: (Protein OR peptide) AND (“in silico” OR “computer simulation”) AND (“molecular dynamics simulation” OR “molecular dynamics” OR “molecular docking simulation”) AND (obesity) Diabetes *Mellitus*: (Protein OR peptide) AND (“in silico” OR “computer simulation”) AND (diabetes)

**Figure 1.**
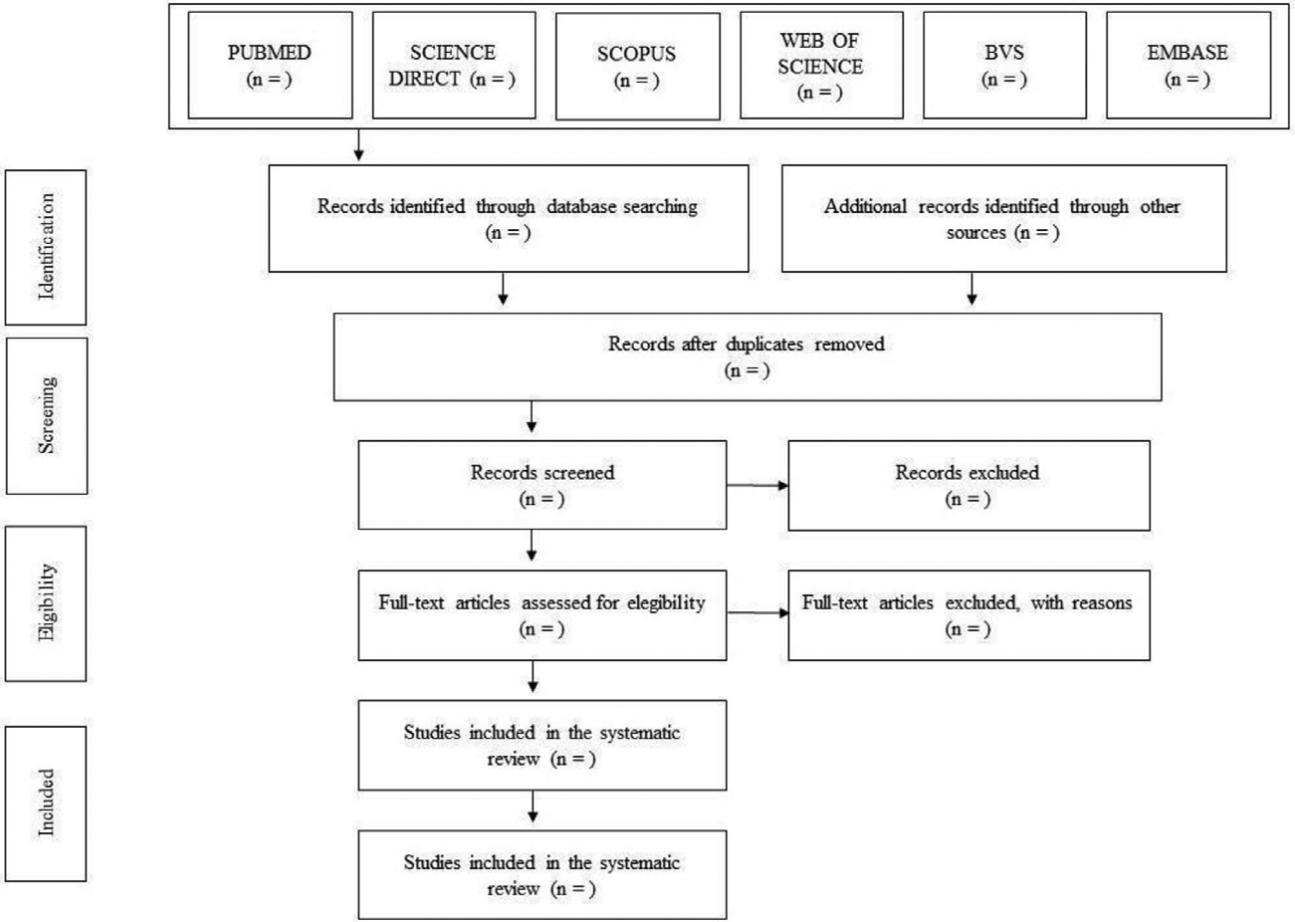
Article selection flowchart adapted from Preferred Reporting Items for Systematic Reviews (PRISMA-P).

The works will be screened by 2 researchers independently. Initially, the titles and abstracts will be read. Subsequently, selected works will be read in full. The references of the included articles will also be reviewed to identify those potentially eligible studies not found in the database search, considered as a manual search (Fig. 1). A third reviewer will resolve disagreements.

### 2.5. Date extraction

After data bases searching and sorting, all articles will be imported into the Rayyan app (version 0.1.0). Migrating articles to this platform will facilitate the removal of duplicate studies and the sorting process (based on inclusion criteria). The following characteristics will be extracted from the selected articles: authors, country, model (in silico), the technique used (docking and molecular dynamics), docking score, interaction potential energy, most interacting amino acids, therapeutic targets, the therapeutic agent used, in vitro/in vivo effects, potential applications, and other timely data. These data will be tabulated in a predefined table.

### 2.6. Risk of bias and quality assessment

The modeling quality will be assessed using a checklist designed based on the standardized guidelines for simulation “Strengthening the reporting of empirical simulation studies” (adapted). The risk of bias, due to the lack of a standardized tool specifically for this type of study, will be assessed based on a checklist obtained from separate literature sources, such as the checklist developed by Taldaev et al^[26]^

### 2.7. Strategy for data synthesis

Data will be summarized through a narrative approach. The reviews will be structured around the type of peptides or proteins in treating obesity or diabetes investigated through in silico simulations.

Summaries of the results and the protocols used in the included studies will be provided in both reviews. The data will be presented in summary tables and narrative reporting to describe the characteristics of the included studies. These data will be presented according to the type of therapeutic targets related to treating obesity/diabetes, as well as 3-dimensional models of these molecules or compounds used in interactions by computer simulations.

## 3. Discussion

Diabesity, characterized by the development of DM associated with obesity, is a global epidemic with great public health costs and severe economic impacts.^[6]^ This chronic noncommunicable disease epidemic is complex, long-lasting, and result from genetic, physiological, environmental, and behavioral factors, some of which can be modified by adopting a healthy lifestyle with physical activity and proper nutrition.^[27,28]^

Other strategies for treating obesity and DM involve using drugs that act on target molecules, such as dipeptidyl peptidase IV, tyrosine phosphatase 1B, AMP-activated protein kinase, interleukin 6, chemokine ligand 2, and pancreatic lipase. Among these, continuous treatments for DM with the use of sitagliptin, vildagliptin, saxagliptin, and metformin, are known to produce side effects such as hypoglycemia, lactic acidosis, gastrointestinal disturbances, weight gain, kidney failure, diarrhea, and others.^[29]^ The treatment of obesity, in turn, has orlistat, the combination of naltrexone/bupropion, liraglutide, and semaglutide. The possible side effects of these drugs are: allergic reactions such as hives, bowel urgency and stomach pain, difficulty breathing, diarrhea or constipation, nausea, vomiting, oily stools, swelling of the face/ throat/ tongue, rectal pain, increased blood pressure, and others.^[30,31]^

An important alternative to traditional medicines that has stood out is the use of molecules from natural or even synthetic sources, such as proteins and peptides, which are gaining more and more space, given the potential to treat chronic noncommunicable diseases. These can act on the same target molecules of classic drugs, thus being able to exert their therapeutic effect, causing fewer side effects.^[20]^

Furthermore, in silico, in vitro and/or in vivo studies show that natural proteins, such as those present in milk,^[13,14]^ soy,^[15]^ wheat,^[32]^ yam,^[33]^ cocoa seed,^[20]^ sesame,^[34]^ plus their respective peptides generated from computational models, show diverse bioactivities regarding the control and treatment of obesity and DM.^[20,34,35]^

In this way, through in silico studies, the most varied interactions have been identified by simulation of molecular dynamics or molecular docking and, with this, driven the discovery of proteins and peptides with therapeutic potential for both obesity and DM. Molecular dynamics simulation allows probing complex processes, such as protein folding, ligand dissociation, and countless other processes, generating diverse data.^[36]^ Molecular docking is usually used to search for interactions between ligands and receptors and can predict their binding mode.^[37]^ Furthermore, they provide data on quantitative structure-activity relationship, which, in terms of discovering bioactive peptides, provides the sequence of the amino acids of these peptides and enables visualization of binding affinity and activity beyond can predict the inhibitory potency of these molecules.^[21]^

Accordingly, computational modeling studies have been widely used in scientific investigations to predict the biological activity, toxicity, pharmacokinetics, and synthesis strategy of compounds based on the structure of the molecule.^[26]^ There is already the prediction of systematic reviews based on protocol focusing on therapeutic targets in silico structure-based design. According to Gomes et al^[38]^, the reviews developed from that protocol will guide decision-making regarding the choice of targets/models in future research focused on therapeutics of obesity or diabetes mellitus. However, therapeutic agents such as peptides and proteins investigated through in silico studies can be promising molecules in generating new drugs, and there is no yet publication with this approach.

Therefore, this protocol will guide the development of 2 systematic reviews to gather information on proteins and/or peptides involved in obesity or diabetes mellitus evaluated in in silico studies. The results could direct future studies contributing to the choice of promising bioactive proteins and/or peptides and the selection of 3-dimensional structures. Therefore, they are potentially effective and less aggressive treatments, in addition to presenting a potentially more positive cost-benefit ratio, which can significantly contribute to the health of populations.

## Author contributions

**Conceptualization:** Ana Heloneida de Araújo Morais.

**Data curation:** Isaiane Medeiros, Ana Júlia Felipe Camelo Aguiar, Juliana Kelly da Silva Maia, Ingrid Wilza Leal Bezerra, Grasiela Piuvezam, Ana Heloneida de Araújo Morais.

**Formal analysis:** Isaiane Medeiros, Ana Júlia Felipe Camelo Aguiar, Juliana Kelly da Silva Maia, Ingrid Wilza Leal Bezerra, Grasiela Piuvezam, Ana Heloneida de Araújo Morais.

**Investigation:** Isaiane Medeiros, Ana Júlia Felipe Camelo Aguiar, Ana Francisca Teixeira Gomes, Wendjilla Fortunato de Medeiros, Emilly Guedes Oliveira e Silva.

**Methodology:** Isaiane Medeiros, Ana Júlia Felipe Camelo Aguiar, Ana Francisca Teixeira Gomes, Wendjilla Fortunato de Medeiros, Grasiela Piuvezam.

**Project administration:** Ana Heloneida de Araújo Morais.

**Supervision:** Ana Heloneida de Araújo Morais.

**Validation:** Isaiane Medeiros, Ana Júlia Felipe Camelo Aguiar, Grasiela Piuvezam.

**Writing – original draft:** Isaiane Medeiros, Ana Júlia Felipe Camelo Aguiar, Juliana Kelly da Silva Maia, Ingrid Wilza Leal Bezerra, Grasiela Piuvezam, Ana Heloneida de Araújo Morais.

**Writing – review & editing:** Isaiane Medeiros, Ana Júlia Felipe Camelo Aguiar, Ana Francisca Teixeira Gomes, Wendjilla Fortunato de Medeiros, Emilly Guedes Oliveira e Silva, Juliana Kelly da Silva Maia, Ingrid Wilza Leal Bezerra, Grasiela Piuvezam, Ana Heloneida de Araújo Morais.
